# Prevalence of and factors associated with hypertension, diabetes, stroke and heart attack multimorbidity in Botswana: Evidence from STEPS 2014 survey

**DOI:** 10.1371/journal.pone.0265722

**Published:** 2022-03-24

**Authors:** Nchidzi Ntiyani, Gobopamang Letamo, Mpho Keetile

**Affiliations:** Department of Population Studies, University of Botswana, Gaborone, Botswana; Yamaguchi University: Yamaguchi Daigaku, JAPAN

## Abstract

**Background:**

Botswana, like other Sub-Saharan Africa (SSA) countries is currently undergoing demographic and epidemiological transitions which are shown by an increase in chronic non-communicable diseases (NCDs) and their associated risk factors. The aim of this study was to examine the prevalence of and factors associated with hypertension, diabetes and stroke/heart attack multimorbidity in Botswana. The definition of multimorbidity used in this study is the presence of two or more NCDs in an individual.

**Methods:**

This study used secondary data derived from the Botswana WHO STEPS 2014 survey. The survey employed a nationally representative multi-stage sampling design. The study sample consisted of 3527 respondents aged 20–69 years of age who had successfully completed the questionnaire and met the inclusion criteria. Multivariable logistic regression analyses were used to assess factors associated with multimorbidity. All comparisons were considered to be statistically significant at 5% level. Statistical tests were performed using Statistical Package for Social Sciences (SPSS) version 25.

**Results:**

Prevalence of hypertension, diabetes and stroke/heart attack multimorbidity was estimated to be at 3.5% in the sampled population. The odds of reporting multimorbidity were highest among females (AOR = 9.73, 95% CI = 8.30–11.42) than males and among respondents aged 35–49 (AOR = 1.20, 95% C.I. = 1.10–1.31) and 50–69 years (AOR = 1.52, 95% C.I. = 1.23–1.67) than individuals aged 20–24 years. Moreover, the odds of multimorbidity were significantly higher among married (AOR = 15.92, 95% C.I. = 13.40–18.92) and living together (AOR = 6.68, 95% C.I. = 5.72–7.81) couples; and individuals who reported that they earn an average annual household income of BWP ≥20 000 (AOR = 2.25, 95% CI = 1.84–2.75) compared to their counterparts. Behavioural risk factors significantly associated with higher odds of multimorbidity were obesity (AOR = 6.79, 95% C.I. = 6.20–7.90), physical inactivity (AOR = 4.41, 95% C.I. = 3.65–5.31) and hazardous alcohol consumption (AOR = 1.49, 95% CI = 1.23–1.81). On the other hand the odds of reporting multimorbidity were significantly low among individuals with sufficient consumption of fruits and vegetables (AOR = 0.47, 95% C.I. = 0.39–0.56) and non-tobacco users (AOR = 0.58, 95% CI = 0.49–0.68).

**Conclusion:**

Multimorbidity was more common among females, the elderly people and was associated with obesity, poor fruit and vegetable intake, and tobacco use. Strategies to combat NCDs and multimorbidity should be aimed to target early stages of life since behavioural factors and lifestyles that increase the likelihood of disease are entrenched in earlier stages of life.

## Introduction

Non-communicable diseases (NCDs) have become a major challenge in global health and their burden has increased in recent years overtaking that of communicable diseases in some countries. NCDs are now the leading cause of death globally, claiming 41 million lives annually, an equivalent to 71% of all deaths globally [1]. According to the World Health Organization (WHO) projections, the total annual number of deaths from NCDs will increase to 55 million by 2030 if current trends are not reversed, and the greatest increases will occur in Africa, South East Asia and the Eastern Mediterranean [1]. NCDs and their risk factors’ prevalence show an increasing trend in low and middle income countries (LMICs). The largest proportion of the NCD burden in LMICs is represented by cardiovascular diseases (CVD), followed by cancer, diabetes and chronic respiratory disease [2]. Evidence has shown that the disparities in health and life expectancy between developing and developed countries, especially in relation to NCDs are due to absence of organised health systems [3].

Global prevalence estimates of multimorbidity ranged from 12.9% (in the general population) to 95.1% (among people 65 years and older) in 2014 [4]. Research evidence shows a rising trend in the prevalence of multimorbidity in the LMICs. Demographic changes, especially population aging contributes to the development of multimorbidity of chronic conditions [5]. Previous studies indicate that multimorbidity is also socially patterned, where the highest prevalence is often observed among populations with socioeconomic deprivations than their wealthier counterparts in high income countries (HICs) [6]. Lifestyle factors such as obesity, physical inactivity, harmful use of alcohol, and psychosocial factors, especially negative life experiences and believing in external locus of control are also factors associated with multimorbidity [7]. Meanwhile, the greatest impact of multimorbidity might even further increase in LMICs where health systems are overwhelmed by the burden of communicable diseases (especially, HIV, TB and Malaria) and maternal, neonatal and nutritional health problems [8]. In 2015, NCDs accounted for 34% of all deaths in sub-Saharan Africa (SSA) [9].

Botswana is currently undergoing demographic and epidemiological transitions hence experiencing an increase in NCDs. It was estimated that 46% of deaths in Botswana were due to NCDs in 2018 [1] which was higher than the NCD deaths in the SSA region. Cardiovascular diseases and diabetes are estimated to cause 18% and 6% of mortality in Botswana respectively, while hypertension was responsible for 8.9% of out-patient morbidity [10, 11]. Research suggests that the majority of patients with NCDs do not have a single diagnosis, but numerous diagnoses coexisting within one person, a scenario referred to as multimorbidity [12]. Multimorbidity subject people to poor health outcomes, high healthcare utilisation and increased cost of care [13–15]. Despite increased multimorbidity, prevalence and determinants studies have remained single disease focused, while most studies on multimorbidity have focused on older populations in high income HICs.

The identification of the determinants of multimorbidity has become a prerequisite for the development of effective strategies for the early identification of patients at risk and for the prevention of future health conditions [16]. Meanwhile, little is known about multimorbidity in Botswana, especially diabetes, hypertension, stroke/heart attack multimorbidity. As a result, there is dearth of information on the risk factors and determinants of multimorbidity. It is against this background that this study sought to assess prevalence of and factors associated with hypertension, diabetes and stroke/heart attack multimorbidity. This study will have significant policy implications by identifying potential population groups at risk of multimorbidity and hence prompting the design of appropriate interventions and strategies aimed at individual, community and national level.

## Materials and methods

### Study design, setting and sampling

This study used a cross-sectional secondary data derived from the latest Botswana WHO STEPS survey conducted in 2014. The survey used the WHO-STEPSwise approach [12]. The STEPS approach is a sequential process that starts with gathering key information on non-communicable disease risks factors with a questionnaire (STEPS 1), simple physical measurements (STEPS 2) and collection of blood samples for biochemical analysis (STEPS 3) [12].

The Botswana WHO STEPS survey was a population based survey of people aged 15–69 years. The survey adopted the 2011 population and housing census sampling frame and employed a multi-stage sampling procedure. A sample size of 300 Enumeration Areas (EAs) was systemically drawn. After identifying EAs households were listed against identified EAs and the proportion of participants was calculated from the total sample size required. Then a computer generated random number was drawn for specific households. All eligible participants residing in the household were listed into handheld data assistant. Finally, a name was picked randomly to participate in the survey. A nationally representative sample of 4074 participants aged 15–69 years took part in the survey [12]. Out of the 4074 participants, 3853 participants responded to at least one of the questions on the presence of NCD (hypertension, diabetes or heart attack/stroke), yielding a response rate of 95%. For this study, we applied a cut off age of 20 years given that multimorbidity was not observed among respondents under the age of 20 years. Hence the 326 participants under the age of 20 years were dropped from this study. The final sample used in the analysis therefore was 3527 participants aged 20–69 years.

### Measures

The outcome variable for this study is hypertension, diabetes or stroke/heart attack multimorbidity. This variable was derived from the list of three NCDs collected during the survey. The definition of multimorbidity used in this study is the presence of two or more NCDs in an individual. During the survey, information was only collected for three NCD conditions; hypertension, diabetes and stroke/heart attack. Participants were asked the following questions;

1. Have you been diagnosed with hypertension in the past 12 months prior to the survey?, Possible responses were, yes & no.
2. Have you been diagnosed with diabetes in the past 12 months prior to the survey? Possible responses were, yes & no.
3. Have you been diagnosed with stroke/heart attack in the past 12 months prior to the survey? Possible responses were, yes & no.

A composite variable was then created such that if an individual reported that he/she was diagnosed with two or more of any of the conditions above, a code of 1 was given while 0 was given if the individual was diagnosed with one or no NCD condition. The final variable was coded into a binary outcome such that ‘1’ meant the presence of multimorbidity and ‘0’ otherwise.

The independent variables were selected with the guide from the Commission on Social Determinants of Health model (CSDH). The CSDH model groups and differentiates the determinants of health into two groups being structural and intermediary determinants to explain correlates of health and wellbeing. Structural determinants generate stratification in the society which in turn influences or defines individual’s social economic position within hierarchies of power, prestige and access to resources and include income, education, occupation, social class, sex, race/ethnicity which determine access to health care [17]. Intermediary determinants of health include material circumstances of living and working conditions and food availability, biological, behavioural and psychosocial factors and the health system itself. These intermediary determinants act as a pathway through which socio-economic position influences health [17]. Therefore based on the CSDH model and data available on the dataset, the structural variables included were age, sex, education level, ethnicity, marital status, employment status and annual household income.

The intermediary variables were tobacco smoking, hazardous alcohol consumption, vegetable intake, fruit intake, obesity, and physical activity. All these variables were self-reported, except for overweight/obesity which was objectively measured through anthropometric information. The variables were measured following the criterion below;

#### Current tobacco use

The respondents were asked if they currently use tobacco products daily. The answers were coded such that “1” denoted “Yes” and “2” denoted “No”.

#### Hazardous alcohol consumption

From the survey the respondents were asked ‘during each of the past 7 days, how many standard drinks did you have each day”? A standard drink contains approximately 10g of pure alcohol. Hazardous alcohol consumption was defined as 40–59.9 g (equivalent to 4–6 drinks) of pure alcohol on average per day for men and 20–39.9g (≥2–4 drinks) for women. Therefore in this study, three or more drinks per day for both sexes were coded 1 to denote hazardous drinking while 2 denoted otherwise.

#### Fruit and vegetable consumption

This variable was computed from two different variables. Firstly, respondents were asked if they eat fruits at least once a week. The respondents who responded affirmatively were further asked how many servings of fruits they eat on one of the days in a typical week. Then the respondents were asked how many servings of vegetables they eat on one of the days in a typical week. For clarification on the definition of a serving, flash cards with depiction and definition of serving were shown to respondents. The use of flash cards was used for respondents to understand equivalence of a serving. The two variables on fruit consumption and vegetable consumption were combined to make the variable fruit and vegetable consumption. The variable was recoded into categorical variable with 1 denoting less than 5 servings (insufficient consumption) and 2 denoting 5 or more servings (sufficient consumption) following WHO guidelines for nutrition [9].

#### BMI

BMI is a continuous variable calculated as weight in kg divided by height in metres squared. This variable was recoded into categories, such that “1” denoted underweight (BMI<18.5), “2” normal weight (18.5≤BMI≤24.9), “3” overweight (25≤BMI≤29.9) and “4” obese (BMI≥30).

#### Physical activity

Respondents were asked whether their work involve vigorous-intensity activity that causes large increases in breathing or heart rate like [carrying or lifting heavy loads, digging or construction work] for at least 10 minutes continuously. All respondents were also asked whether they walk or use a bicycle (pedal cycle) for at least 10 minutes continuously to get to and from places with binary answers being 1-yes and 2- No. In addition to that, they were asked whether they do any vigorous-intensity sports, fitness or recreational (leisure) activities that cause large increases in breathing or heart rate like [running or football] for at least 10 minutes continuously with answers being 1-yes and 2-No.

Based on the WHO recommendation on the activity for work, transport and recreational time, an adult should throughout a week do at least 150 minutes of moderate-intensity physical activity, or 75 minutes of vigorous-intensity physical activity or an equivalent combination of moderate and vigorous-intensity physical activity of at least 600 MET-minutes. An individual doing less than 150 minutes of moderate-intensity physical activity is regarded physical inactive. In this study MET-minutes per week were used to measure physical activity or inactivity. MET values were assigned to each activity and then multiplied by the number of days in a week on which the activity was performed and then multiplied by the minutes taken doing the activity per day, to get the new variable MET-minutes per week. MET values were calculated for each activity.

The sum of the MET-minutes per week was used to create Total MET-minutes per week. From this a final binary variable was derived based on Total MET-minutes per week; namely ‘Physical activity’ denoted by “2” ≥600 MET-minutes per week and ‘Physical inactivity’ denoted by “1” <600 MET-minutes per week.

### Statistical analysis

There were three levels of statistical analyses required to meet the objectives of this study. Univariate analyses through the generation of simple frequencies and the use of percentage distributions were performed to determine the prevalence of multimorbidity. Bivariate analysis was conducted to analyse the patterns of multimorbidity among males and females. The last level of analysis was multivariate logistic regression analyses which were used to examine correlates of multimorbidity. The level of analysis was carried out at 95% significance level.

Four models of binary logistic regression were used. Model 1 presented the unadjusted or crude analysis of possible determinants of multimorbidity. In this model the results were not adjusted for the effect of confounding factors. Model 2 or intermediary model represented a binary logistic regression of intermediary variables and multimorbidity only as per the CSDH conceptual model. The adjusted variables in this model are BMI, physical activity, fruit and vegetable consumption, tobacco use and hazardous alcohol consumption. Model 3 or structural model represented a multivariate binary logistic regression of structural variables and multimorbidity only adjusting for the effect of sex, age, education level, nationality, marital status, work status and annual income, while the final model (Model 4) was the adjusted analysis of all variables. Prior to conducting the multivariable logistic regression analyses, a multi-colliniarity test was carried out among all the statistically significant variables to determine if there was evidence of multi-colliniarity between them. Using the variance inflation factor (VIF), the multicolliniarity test showed that there was no evidence of colliniarity among the explanatory variables. Cases with missing values for particular variables were not included in the analysis. This assumes data are missing completely at random (MCAR).

The association between the dependent and independent variables was examined simultaneously adjusting for possible confounding. Statistical tests were performed using Statistical Package for Social Sciences (SPSS) version 25. Given that the multi-stage stratified sampling design was used during the STEPS 2014 we used complex sample module in SPSS in order to account for the hierarchical structure of the sampling design.

### Ethical considerations

This paper is based on the analysis of secondary data. Since the data used in this study was collected using the WHO STEPWise approach and WHO standard questionnaires all the instruments used in this study were validated and approved by the WHO and Ministry of Health and Wellness in Botswana. Our study employed secondary data analysis, and there were no ethical issues of concern for this paper. All ethical issues were addressed at the time of data collection during the survey by the Ministry of Health and Wellness (MoHW) and WHO.

## Results

### Socio-demographic characteristics of the sample

The results in Table 1 show that a large proportion of the sample was accounted for by females (67.3%). The age distribution indicates that majority of the sampled population were less than 50 years of age (77%). Just over one third of respondents (34.3%) had primary or less education, under half (46.8%) had secondary education while just below a fifth (18.9%) had tertiary or higher education level. Meanwhile, about three fifths (61.7%) of the respondents were never married, while just over a quarter (25.6%) had an annual income range between BWP 5,000—BWP 9,999.

**Table 1:** Weighted socio economic and demographic characteristics of the study population.

Variable	Frequency (N)	Percentage (%)
**Sex**		
Male	1154	32.7
Female	2373	67.3
**Age in years**		
20–34	1644	46.6
35–49	1073	30.4
50–69	810	23.0
**Educational level**		
Primary or less	1210	34.3
Secondary	1650	46.8
Tertiary or Higher	667	18.9
**Nationality**		
Batswana	3398	96.4
Other nations	128	3.6
**Marital status**		
Never married	2174	61.7
Married	739	21.0
Separated/Divorced/ widowed	228	6.5
Living together	381	10.8
**Work status**		
Government employees	538	15.3
Parastatal/NGO employees	496	14.1
Self employed	464	13.2
Non paid/unemployed	2027	57.5
**Annual income in BWP**		
<5000	601	22.6
5000–9999	681	25.6
10000–14999	336	12.6
15000–19999	227	8.5
≥20000	815	30.6
**BMI**		
Underweight	396	11.2
Normal	1655	47.0
Overweight	833	23.6
Obesity	639	18.1
**Physical activity**		
Physical inactive	1011	28.7
Physical active	2516	71.3
**Fruit & vegetable consumption**		
Insufficient	1815	82.6
Sufficient	382	17.4
**Tobacco use**		
Yes	486	13.8
No	3041	86.2
**Hazardous alcohol consumption**		
Yes	117	17.1
No	569	82.9
**Total**	**3527**	**100.0**

The proportion of respondents who were overweight, obese, and were physical inactive was 23.6%, 18.1%, and 28.7%, respectively. On the other hand the proportion of respondents who reported insufficient fruit and vegetable consumption was 82.6%, while 13.8% reported tobacco use and 17.1% were hazardous alcohol consumers.

### Prevalence of hypertension, diabetes, hypertension, diabetes or stroke/heart attack multimorbidity

**Fig 1:**
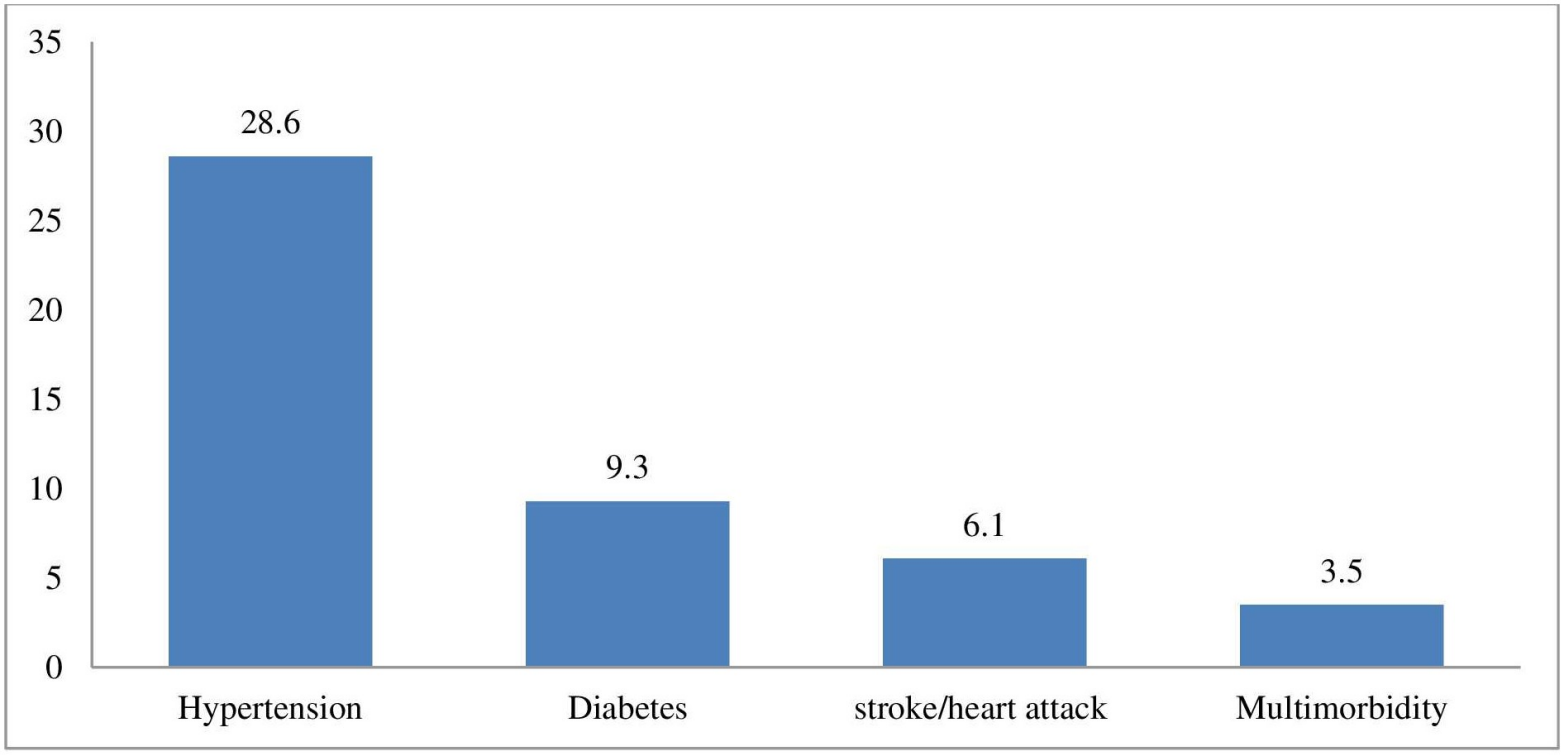
Prevalence of hypertension, diabetes or stroke/heart attack and multimorbidity.

Fig 1 show that the prevalence of hypertension diabetes, stroke/heart attack and multimorbidity among adults aged 20–69 years was estimated to be 28.6%, 9.3%, 6.1% and 3.5% respectively.

The prevalence of hypertension, diabetes or stroke/heart attack multimorbidity was high among females (4.1%) and increased with age, with just over a tenth of both males (10.2%) and females (10.3%) aged 50–69 years reporting multimorbidity (Table 2). Multimorbidity was also highest among males with tertiary or higher education (5.4%) and females with primary or less education (6.2%), and separated/divorced/widowed respondents (males = 14.4% vs females = 9.4%). Multimorbidity increased with BMI, with highest prevalence noted among obese males (19.5%) and females (8.6%). Surprisingly, multimorbidity was slightly more prevalent among females (5%) who were physically active but less prevalent among physically inactive males (4%). The prevalence of multimorbidity was highest among females who reported insufficient consumption of fruits and vegetables (4.7%). Quite conversely, multimorbidity was lowest among males (1.9%) and females (1.7%) who reported tobacco smoking.

**Table 2:** Prevalence and patterns of hypertension, diabetes, stroke/heart attack and multimorbidity stratified by sex.

Variable	Hypertension		Diabetes		Stroke/heart attack		Multimorbidity	
	Male (n = 922)	Female (n = 2112)	Male (n = 287)	Female (n = 716)	Male (n = 1154)	Female (n = 2372)	Male (n = 1154)	Female (n = 2372)
	%	%	%	%	%	%	%	%
**Age in years**								
20–34	16.8	22.2	2.5	4.5	4.0	5.6	0.8	1.6
35–49	29.9	36.4	12.8	9.0	4.4	8.4	3.6	4.2
50–69	40.5	55.2	19.4	13.3	9.4	11.0	10.2	10.3
**Educational level**								
Primary or less	30.8	44.4	12.3	10.3	4.2	11.1	3.4	6.2
Secondary	18.8	28.8	6.1	8.1	4.2	5.6	1.4	3.2
Tertiary or Higher	26.4	25.3	11.8	8.1	7.2	6.6	5.4	3.8
**Nationality**								
Batswana	24.1	32.9	8.9	8.6	5.0	7.6	2.5	4.3
Other nations	27.0	44.7	20.7	22.7	3.7	0.8	8.9	1.2
**Marital status**								
Never married	18.3	27.8	3.4	6.4	3.6	6.4	0.6	2.9
Married	39.3	41.3	21.1	10.0	6.8	8.4	12.1	7.6
Separated/Divorced/ widowed	38.6	47.7	14.7	19.9	9.0	11.8	14.4	9.4
Living together	28.9	34.9	4.8	3.4	9.8	8.3	1.5	2.2
**Work status**								
Government employees	33.2	33.4	8.0	11.4	8.2	8.2	4.9	7.5
Parastatal/NGO employees	21.4	36.6	5.0	7.8	4.8	8.5	3.2	3.9
Self employed	26.6	41.6	17.7	14.7	4.1	1.4	3.7	6.8
Non paid/unemployed	21.8	30.2	9.5	6.5	4.3	7.8	1.7	3.2
**Annual income in BWP**								
<5000	15.0	20.0	28.1	12.5	16.8	24.4	4.3	4.0
5000–9999	15.8	26.0	1.6	14.2	18.0	35.5	0.3	3.2
10000–14999	8.2	11.6	8.0	7.5	16.5	7.7	3.8	1.8
15000–19999	12.2	6.2	8.3	22.0	20.7	6.1	3.5	3.7
≥20000	48.8	36.2	53.9	43.8	28.1	26.3	3.4	7.3
**BMI**								
Underweight	13.7	16.9	-	-	4.0	6.7	0.8	0.7
Normal	22.6	24.8	8.2	3.6	4.6	8.5	1.4	2.5
Overweight	32.1	38.1	9.3	11.1	3.8	5.7	4.9	4.9
Obesity	47.2	48.4	23.8	13.4	12.3	7.1	19.5	8.6
**Physical activity**								
Physical inactive	27.6	36.1	13.0	9.2	3.3	3.7	4.0	2.2
Physical active	23.2	31.4	8.8	8.6	5.3	8.8	2.7	5.0
**Fruit & vegetable consumption**								
Insufficient	25.6	36.4	8.8	10.7	4.4	5.6	3.2	4.7
Sufficient	30.0	38.1	12.6	16.5	4.1	3.4	4.2	4.6
**Tobacco use**								
Yes	25.8	26.0	9.5	7.6	5.5	14.6	1.9	1.7
No	23.5	33.2	9.8	8.8	4.5	6.8	3.5	4.3
**Hazardous alcohol consumption**								
Yes	23.6	23.3	5.3	13.5	5.0	10.3	1.3	-
No	15.9	48.5	-	-	3.3	11.4	1.5	4.7
**Overall %**	**24.7**	**32.5**	**9.6**	**9.0**	**5.2**	**7.0**	**2.9**	**4.1**

### Correlates of multimorbidity

The association between socio-demographic, behavioural factors and hypertension, diabetes or stroke/heart attack multimorbidity is shown in Table 3. In the adjusted model sex was found to be significantly associated with multimorbidity, with females 9 times more likely (AOR = 9.73, 95% C.I. = 8.30–11.42) to report multimorbidity than males when adjusting for other socio-demographic and behavioural factors. Age was also found to be associated with multimorbidity. For instance, elderly people aged 50–69 years were more likely (AOR = 1.52, 95% C.I. = 1.23–1.67) to report multimorbidity than younger people aged 20–34 years. The odds of reporting multimorbidity were observed to be 15 times higher (AOR = 15.92, 95% C.I. = 13.40–18.92) among married respondents than those never married. Self-employed and non-paid/unemployed respondents were 52% (AOR = 0.48, 95% C.I. = 0.45–0.51) and 58% (AOR = 0.42, 95% C.I. = 0.40–0.44) less likely to report multimorbidity than government employees, respectively. Individuals with household income of BWP 20 000 or more were also more likely (AOR = 2.25, 95% C.I. = 1.84–2.75) to report multimorbidity compared to those with household income of less than BWP 5,000 when adjusting for other factors.

**Table 3:** Logistic regression analysis results of factors associated with hypertension, diabetes or stroke/heart attack multimorbidity.

Variable	Model 1-Unadjusted OR (95% CI)	Model 2-Intermediary OR (95% CI)	Model 3- Structural OR (95% CI)	Model 4—AOR (95% CI)
**Sex**				
Male	1		1	1
Female	1.45*** (1.41–1.49)		1.78***(1.72–1.84)	9.73***(8.30–11.42)
**Age in years**				
20–34	1		1	1
35–49	3.89*** (3.74–4.05)		2.34*** (2.24–2.46)	1.20*** (1.10–1.31)
50–69	9.59*** (9.24–9.95)		6.89*** (6.53–7.28)	1.52*** (1.23–1.67)
**Educational level**				
Primary or less	1		1	1
Secondary	0.49*** (0.47–0.50)		1.19*** (1.13–1.24)	0.73*** (0.61–0.86)
Tertiary or Higher	1.02 (0.98–1.05)		0.84*** (0.80–0.89)	0.06*** (0.04–0.08)
**Nationality**				
Batswana	1		1	1
Other nations	2.03*** (1.94–2.14)		2.26*** (2.13–2.39)	0.47*** (0.37–0.59)
**Marital status**				
Never married	1		1	1
Married	6.30*** (6.10–6.50)		1.78*** (1.71–1.86)	15.92*** (13.40–18.92)
Separated/Divorced/ widowed	7.32*** (7.00–7.66)		1.46*** (1.38–1.55)	1.68 (0.73–1.78)
Living together	1.10*** (1.04–1.18)		0.75*** (0.70–0.81)	6.68*** (5.72–7.81)
**Work status**				
Government employees	1		1	1
Parastatal/NGO employees	0.50*** (0.48–0.52)		0.81*** (0.77–0.86)	0.13 (0.08–1.82)
Self employed	0.71*** (0.68–0.74)		0.48*** (0.45–0.51)	0.48*** (0.40–0.56)
Non paid/unemployed	0.36*** (0.35–0.38)		0.42*** (0.40–0.44)	0.81*** (0.70–0.94)
**Annual income in BWP**				
<5000	1		1	1
5000–9999	0.43*** (0.40–0.45)		0.44*** (0.42–0.47)	1.03 (0.85–1.33)
10000–14999	0.65*** (0.61–0.69)		0.68*** (0.63–0.72)	5.03*** (3.98–6.36)
15000–19999	0.86*** (0.81–0.92)		0.99 (0.93–1.06)	0.84 (0.63–1.14)
≥20000	1.25*** (1.21–1.30)		1.06*** (1.01–1.12)	2.25*** (1.84–2.75)
**BMI**				
Underweight	1	1		1
Normal	2.50*** (2.31–2.71)	1.25*** (1.10–3.30)		1.09 (0.98–1.54)
Overweight	6.78*** (6.23–7.35)	3.49*** (2.01–4.75)		2.64*** (1.60–2.99)
Obesity	16.38*** (15.13–17.74)	6.26*** (5.51–7.08)		6.79*** (6.20–7.90)
**Physical activity**				
Physical active	1	1		1
Physical inactive	0.77*** (0.75–0.80)	1.87*** (1.68–2.07)		4.41*** (3.65–5.31)
**Fruit & vegetable consumption**				
Insufficient	1	1		1
Sufficient	1.07 (1.02–1.11)	0.60*** (0.54–0.67)		0.47*** (0.39–0.56)
**Smoking**				
Yes	1	1		1
No	2.16*** (2.07–2.26)	3.23*** (2.91–3.59)		0.58*** (0.49–0.68)
**Hazardous alcohol consumption**				
No	1	1		1
Yes	2.16*** (1.91–2.44)	0.49*** (0.43–0.55)		1.49*** (1.23–1.81)

***p<0.001;

**p<0.05;

CI- confidence interval, OR-Odd ratios, AOR = Adjusted Odd Ratios, Model 1 = unadjusted model, Model 2 or intermediary model = adjusted for BMI, physical activity, fruit and vegetable consumption, tobacco use and hazardous alcohol consumption. Model 3 or structural model = adjusted for sex, age, education level, nationality, marital status, work status and annual income, Model 4 = Adjusted for sex, age, education level, nationality, marital status, work status, annual income, BMI, physical activity, fruit and vegetable consumption, tobacco use and hazardous alcohol consumption.

For behavioural factors, multimorbidity was 6 times higher among respondents with obesity (AOR = 6.79, 95% C.I. = 6.20–7.90) than among those with underweight. Respondents who were physical inactive were more likely to report multimorbidity (AOR = 4.41, 95% C = 3.65–5.31) than those who were physical active. Fruit and vegetable consumption was also significantly associated with multimorbidity after adjusting for other behavioural and socio-demographic factors. Individuals who reported sufficient consumption of fruits and vegetables were 53% less likely (AOR = 0.47, 95% CI = 0.39–0.56) to report multimorbidity compared to those who consumed insufficient servings of fruits and vegetables. Individuals who did not use tobacco products had lower odds of multimorbidity (AOR = 0.58, 95% C.I. = 0.49–0.68) than those who used tobacco products while individuals who reported hazardous use of alcohol had higher odds of multimorbidity (AOR = 1.49, 95% C.I. = 1.23–1.81) than those who did not.

## Discussion

Findings from this study indicate that hypertension, diabetes or stroke/heart attack multimorbidity prevalence in the sampled population was estimated to be 3.5%. The prevalence of hypertension, diabetes and stroke/heart attack observed in this study is comparatively high than for most countries in the region [18]. This makes NCDs a significant public health challenge. The increasing prevalence of NCDs is often linked to coexistence of several NCD conditions in one individual leading to multimorbidity, which is an impending concern in Botswana [19]. The health care system is already beleaguered by communicable diseases, particularly HIV/AIDS [18] and increasing NCDs [19].

Consistent with most studies we found that hypertension, diabetes or stroke/heart attack multimorbidity was more prevalent among older individuals [20, 21]. The prevalence of multimorbidity increased with age and was highest at age 50–69 years. Most studies examining multimorbidity have shown that multimorbidity among the elderly is associated with the cumulative effect of NCD risk factors due to age [21]. Similarly, in this study a high proportion of individuals who were aged 50–69 years reported physical inactivity, hypertension, overweight and obesity, and current tobacco use. The clustering of NCD risk factors in this population group predisposes them to multiple NCD conditions. This explains why the older population in this study reported the highest prevalence of multimorbidity than the younger population group.

We also found that there were sex differentials in multimorbidity with high rates of multimorbidity observed among females than males. This finding conforms to the results of other studies which also found a higher prevalence of multimorbidity among females [22–24]. In both HICs and LMICs evidence suggests that sex differentials are very important in epidemiology and pathophysiology of many diseases especially NCDs [25]. Generally females have been observed to show high propensity to use health services than males, consequently they are more likely to be diagnosed or told that they have NCDs compared to males [26]. Furthermore, the sex differences in multimorbidity observed in this study can also be attributed to the high prevalence of obesity among females than males.

With regards to education level, the results indicated that there was a negative association with multimorbidity with the odds of having multimorbidity decreasing with increase in education level. This finding corroborates previous studies [25–27]. Education is vital in health promotion and empowers people to seek information. Therefore educated people may access more information better, use it to access healthcare and adopt healthy lifestyles, reducing behavioural risk factors and preventing onset of chronic conditions while people with a low level of education may have little awareness of healthy behaviours and difficulties in accessing care [27–29].

The odds of multimorbidity were significantly higher among married than non-married individuals, consistent with findings of one study in Ghana [30]. However; this finding contradicts results from other various studies which did not show any association between marital status and multimorbidity [31]. Consistent with other previous studies annual household income was found to be associated with multimorbidity [22, 23, 27, 32]. More affluent households and communities are argued to adopt sedentary lifestyles and diets which predispose them to chronic conditions.

Consistent with previous studies we found a significant association between BMI and multimorbidity with the odds of multimorbidity six times higher among individuals who were categorised as obese compared to those who were underweight. Research evidence has shown that BMI is an important determinant of NCDs and multimorbidity. Some studies have found that BMI is positively associated with multimorbidity [27, 33]. For instance, a study in South Africa showed that respondents who were obese were more likely to have multimorbidity than those not obese [27]. All intermediary variables (hazardous alcohol consumption, current tobacco use, alcohol consumption, BMI, physical activity, fruit and vegetable intake) were found to be significantly associated with multimorbidity. Intermediary variables are associated with multimorbidity through the clustering effect where multiple NCD risk factors are found to cluster in individuals predisposing them to several NCD conditions [22, 25, 27, 33–38].

Regular physical activity has been found to play an important role in the primary and secondary prevention of NCDs as well as improving quality of life [39, 40]. As expected, this study found that respondents who were physical inactive had higher odds of having multimorbidity than those who were physical active. This is in agreement with a study conducted in six LMICs which found the odds of having multimorbidity were high among respondents who had high sedentary behaviour [41–48]. This reiterates that indeed lack of physical activity is a risk factor for NCDs and multimorbidity.

This study had strengths that are worth mentioning. This was the first study to our knowledge that examined hypertension, diabetes and stroke/heart attack multimorbidity and its correlates in Botswana, and it therefore provides novel information for policy and programme intervention. Meanwhile this is study has the following limitations; this study used a cross-sectional study design, limiting the study to associations and cannot infer causal link. Since the study relied on secondary data it was not possible to explore other important variables on multimorbidity such as social class, political context, and psychosocial factors.

## Conclusion

The prevalence of hypertension, diabetes, stroke/heart attack and multimorbidity among adults aged 20–69 years in Botswana in 2014 was 28.6%, 9.3%, 6.1% and 3.5%, respectively. Sex differentials were found for the prevalence of the three NCDs and their multimorbidity. The prevalence of hypertension, stroke/heart attack- and multimorbidity was highest among females, increased with age, higher education, married individuals, and annual household income. Moreover, the odds of multimorbidity were higher among individuals with obesity, physical inactive, tobacco smokers and those consuming alcohol hazardously. An understanding of factors associated with multimorbidity is necessary for designing strategies aimed at preventing multimorbidity and encourage active and healthy lifestyles. The Botswana multi-sectoral strategy on NCDs should aim at creating innovative and new approaches aimed at preventing the risks of NCDs and multimorbidity such as the investing in the life course approach which considers the critical stages, transitions and settings where differences can be made in promoting or restoring health and wellbeing. For example, enhancing and strengthening school health programmes through provision of healthy food and promotion of physical activity and sports in schools. Furthermore, collaborative effort with relevant stakeholders is imperative to raise awareness and health promotion at individual, community and national level with more emphasis on women. Workplaces should also offer opportunities for healthy food, physical activity and periodic health screening or assessments of employees. Botswana should also strengthen the implementation and enforcement of WHO Framework Convention on Tobacco Control (FCTC), regulate selling and marketing of tobacco products and alcohol. This study used few disease conditions, therefore future research should capture more disease conditions and other possible cofounding variables on diet such as dietary sugar, salt and fat intake and social class, political context, and psychosocial factors. Furthermore, longitudinal and interventional studies are also needed in the future to establish causal relationships.

## Supporting information

S1 File (SAV)
